# Analysis of conjugated steroid androgens: Deconjugation, derivatisation and associated issues

**DOI:** 10.1016/j.jpba.2009.01.027

**Published:** 2009-07-12

**Authors:** Rachel L. Gomes, Will Meredith, Colin E. Snape, Mark A. Sephton

**Affiliations:** a Department of Earth Science and Engineering, Imperial College London, South Kensington, London SW7 2AZ, UK; b Nottingham Fuel and Energy Centre, School of Chemical and Environmental Engineering, University of Nottingham, University Park, Nottingham NG7 2RD, UK

**Keywords:** ^13^C/^12^C, stable carbon isotope ratio, APS, adenosine-5′-phosphate, ATP, adenosine triphosphate, BSTFA, *N*,*O*-bis(trimethylsilyl)-trifluoroacetamide, DHEA, dehydroepiandrosterone, ES, external standard, FID, flame ionisation detector, FU, Fishman unit, G, glucuronide, GC/C/IRMS, gas chromatography/combustion/isotopic ratio mass spectrometry, GC/MS, gas chromatography/mass spectrometry, IS, internal standard, IU, international unit, LC/MS, liquid chromatography/mass spectrometry, L–L, liquid–liquid, MSTFA, *N*-methyl-*N*-(trimethylsilyl)-trifluoroacetamide, PAPS, 3′,5′-phosphoadenosine, P-Pi, pyrophosphate, S, sulfate, SPE, solid phase extraction, T/E ratio, testosterone to epitestosterone ratio, TMCS, trimethylchlorosilane, TMIS, trimethyliodosilane, TMS, trimethylsilyating, U, unit, UDPGA, uridine diphosphoglucuronic acid, RU, Roy unit, WADA, World Anti-Doping Agency, Conjugated steroids, Biological samples, Forensic drug testing, Deconjugation, Derivatisation

## Abstract

Gas chromatography/mass spectrometry (GC/MS) is the preferred technique for the detection of urinary steroid androgens for drug testing in athletics. Excreted in either the glucuronide or sulfated conjugated form, steroids must first undergo deconjugation followed by derivatisation to render them suitable for GC analysis. Discussed herein are the deconjugation and the derivatisation preparative options. The analytical challenges surrounding these preparatory approaches, in particular the inability to cleave the sulfate moiety have led to a focus on testing protocols that reply on glucuronide conjugates. Other approaches which alleviate the need for deconjugation and derivatisation are also highlighted.

## 1 Introduction

**Fig. 1:**
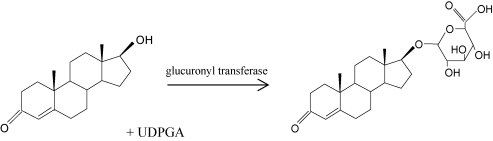
Conjugation of a glucuronide moiety to the free steroid androgen, testosterone [UDPGA, uridine diphosphoglucuronic acid].

**Fig. 2:**
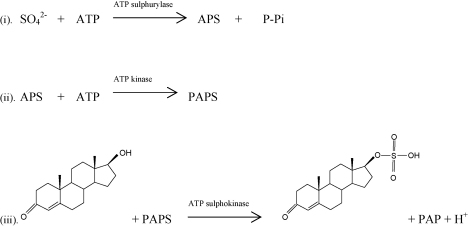
Conjugation of a sulfate moiety to the free steroid androgen, testosterone [PAPS, 3′,5′-phosphoadenosine; P-Pi, pyrophosphate; ATP, adenosine triphosphate; APS, adenosine-5′-phosphate].

The ever increasing desire of certain athletes to enhance their performance with the aid of anabolic and androgen drugs has been met by the analytical chemist's need to identify such cheats. Gas chromatography/mass spectrometry (GC/MS) is the preferred analytical tool for quantifying urinary steroid androgens. However, unresolved issues with determination lie in the preparative steps necessary to enable GC analysis of steroid androgens [1]. To promote urinary excretion, steroids undergo phase II metabolism which increases the hydrophilic nature of the steroid. This is achieved by conjugation with either a glucuronide (Fig. 1) or sulfate (Fig. 2) moiety. Less than 3% of the total androgens excreted via the urine is unconjugated [2,3]. Conjugation of steroid androgens is generally with a glucuronide moiety. However, steroid androgens such as androsterone, etiocholanolone, testosterone and epitestosterone may also be excreted as sulfates, with a sulfate to glucuronide ratio nearing 1:1 in some instances [4,5].

For GC determination, the analyte of interest must be volatile and thermally stable. Since steroid conjugates fulfil neither criterion [6], preparative steps are required to render the conjugated steroid amenable to GC analysis. This entails the conjugated steroid undergoing deconjugation or hydrolysis to produce the free steroid, followed by derivatisation to avoid poor chromatographic performance. There are certain challenges that surround these preparatory approaches, in particular the ability to efficiently cleave the sulfate moiety [7]. Therefore the analytical focus has been on the glucuronide conjugates which are far easier to hydrolyse.

An established list defined by the World Anti-Doping Agency (WADA) documents the exogenous (e.g. nandrolone) and endogenous (e.g. testosterone, dehydroepiandrosterone – DHEA) steroid androgens [8]. An important indicator for steroid abuse is the steroid profile, of which the concentration ratio of testosterone glucuronide to epitestosterone glucuronide (T/E ratio) has been adopted for the detection of testosterone doping using gas chromatography/mass spectrometry (GC/MS). Being a minor product of testosterone metabolism, epitestosterone does not increase after administration of testosterone, so a T/E ratio of ≥6.0 constitutes an offence. However, the threshold can be influenced by several metabolic and external parameters. This has resulted in WADA guidance stating that urine samples should be submitted to gas chromatography/combustion/isotopic ratio mass spectrometry (GC/C/IRMS) if the T/E ratio is ≥4.0, or if concentrations are greater than the set limits for testosterone, epitestosterone, androsterone, etiocholanolone or DHEA (all derived from the glucuronide) [9]. By utilising GC/C/IRMS, stable carbon isotope ratios (^13^C/^12^C) allow discrimination between exogenous and endogenous steroids, the exogenous origin containing less ^13^C than their endogenous homologues.

This paper reviews the options available for deconjugation and derivatisation, along with the issues which have dictated the current direction to steroid androgen analysis in the anti-doping field. Attention is also paid to two other approaches that alleviate the need for the deconjugation and derivatisation steps are also discussed, these being liquid chromatography/mass spectrometry (LC/MS) which allows direct determination of the conjugate and the potential of hydropyrolysis as a preparative technique for GC/C/IRMS determination.

## 2 Deconjugation approaches

As conjugated steroids degrade under the high temperatures required for GC analysis, the first step is deconjugation or hydrolysis of the steroid conjugate to its free form. Hydrolysis can be carried out by biological (enzymatic) or chemical means (non-enzymatic). Enzymatic methods are the approaches most commonly used [10]. There are three main sources of enzymes:

1. Mammalian: extracted from beef liver containing Ketodase,
2. Bacterial: from *Escherichia coli* and,
3. Molluscs: the most commonly used is *Helix pomatia* with lesser used sources from the genera *Patella* (*Patella vulgata*), *Haliotis* (abalone) and *Ampullaria*.

The mammalian and bacterial sources contain β-glucuronidase activity allowing cleavage of the glucuronide moiety. The mollusc sources contain β-glucuronidase and sulfatase activity, the latter responsible for sulfate hydrolysis. Though all enzyme sources possess β-glucuronidase activity, the amount of activity and specificity varies. Ketodase was traditionally used for glucuronide hydrolysis but has since been superseded by *E. coli* which is highly specific to β-glucuronides. To date, testing has focused on glucuronidase-only hydrolysis, as exemplified by the 2004 Summer Olympics held in Athens which utilised *E. coli* [11].

Unfortunately, hydrolysing only the glucuronide conjugates may lead to inaccuracies, as the ratio of glucuronide to sulfate excretion of a steroid is dependent on the athlete's metabolism and degree of conversion to the sulfate conjugate [12,13]. A recent study of the 19-norsteroids identified that the contribution of sulfoconjugates varied depending upon the individual and the drug administered. The following metabolic possibilities were given [14]:

a. The absence of sulfoconjugates may occur in some individuals and at some times during the metabolism,
b. The amount of sulfates may exceed glucuronides during excretion in the kidney,
c. Slower elimination of sulfoconjugates than glucuroconjugates prior to excretion may explain the reversed proportions during later excretion stages and,
d. A delay of phase II metabolism of sulfate conjugates may occur.

Relative proportions of a steroid present as a sulfate conjugate can be enhanced by drug administration. Doping with either 5α-androstane-3β,17β-diol or its precursor dihydrotestosterone leads to marked increases in urinary epiandrosterone sulfate and 5α steroids [15]. The sulfate to glucuronide ratio can also be influenced by external factors such as ethanol consumption [16]. The usefulness of determining sulfate conjugates is demonstrated with the testosterone glucuronide to total epitestosterone (glucuronide and sulfate) ratio which has been proven to aid the discrimination between physiologically high and pharmacologically high T/E glucuronide ratios [5].

Whilst hydrolysing sulfate in addition to glucuronide conjugates is advantageous to detecting doping offences, a widely applicable deconjugation procedure is unavailable. The choice of a general enzyme preparation can be difficult owing to their high specificity (Table 1). The efficacy of enzymatic sulfate hydrolysis can be affected by the carbon positioning of the sulfate moiety and the alpha/beta configuration. *H. pomatia* is the most widely used enzyme preparation containing sulfatase activity and has the broadest specificity. Comparatively, *P. vulgata* possesses weak sulfatase activity which is highly specific for 3β-hydroxy-5α- and -3β-hydroxy-Δ^5^-steroids e.g. epiandrosterone and DHEA. However, neither preparation is able to hydrolyse 17 keto steroids sulfates with 3α- and 5α-configuration such as androsterone-3-sulfate [17–19]. Steric hindrance is absolute when both the sulfate on carbon 3 and hydrogen on carbon 5 are in the alpha position, and some hindrance is observed when both are in the β position [20]. Consequently, the enzymatic hydrolysis of a sulfate conjugate produces only a specific part of the total inventory.

In addition to incomplete sulfate hydrolysis, incubation with *H. pomatia* can also lead to steroid conversion or degradation, and artefact formation. As well as glucuronidase and sulfatase activity, *H. pomatia* contains other enzymes including 3β-hydroxysteriod: NAD oxyreductase and 3-oxosteroid-5-4-ene-isomerase [21]. On incubation with androst-5-ene-3β,17β-diol, these additional enzymes produce a similar series of compounds with a 17-hydroxy function, including testosterone. They are also responsible for transforming DHEA to androst-4-ene-3,17-dione which is a constituent in equine urine, as well as other artefacts [22]. Hence, not only is the concentration of the steroid of interest decreased but the product formed may adversely influence the steroid profile. Approaches to overcome the issues with *H. pomatia* are areas of ongoing study and recently the use of sodium ascorbate during hydrolysis with *H. pomatia* has been shown to improve the steroid yield [23].

As well as deciding on the enzyme preparation, optimisation of the hydrolysis conditions is of vital importance to ensure complete deconjugation. The conditions which influence enzyme hydrolysis are the amount of enzyme, temperature, duration of incubation and the pH. The enzyme activity must be sufficient to enable complete hydrolysis of the conjugates present in a sample. However, the amount of enzyme required can be influenced by the type and concentration of the steroid and the other hydrolysis conditions. Additionally, the selectivity and reactivity of the enzyme preparation can vary depending on the source and/or batch [24]. The enzyme activity expressed as either units, Fishman units or Roy units for a particular preparation has been reported to be less than that stated by the manufacturer [18]. Consequently, numerous authors either test the enzyme activity of the preparation before use or carry out preliminary studies to determine the optimum amount of preparation required for complete hydrolysis. As the concentration of the steroid is obviously unknown, determining the correct amount of enzyme is achievable by either a trial and error approach or using excess enzyme. Unfortunately, the trial and error approach is labour, money and time intensive whilst using excess enzyme can lead to a decrease in hydrolysis efficiency [25].

The choice of temperature for incubation is steroid-dependent. Cleavage of DHEA sulfate is improved at higher temperatures whilst androsterone and etiocholanolone sulfate deconjugation is favoured at lower temperatures [19]. If these three steroids were being assessed together, the sample would have to be divided into aliquots prior to *H. pomatia* hydrolysis as recovery of the latter two steroids is decreased when using the higher temperatures [18]. The incubation duration is typically short for hydrolysis with *E. coli* or Ketodase as cleavage of the glucuronides can occur within minutes [10]. However, a duration of 22 h for *E. coli* has been cited in one recent study for testosterone, epitestosterone, etiocholanolone, epiandrosterone, androsterone and 5α-androstane-3α,17β-diol [26]. For the enzymes possessing glucuronidase and sulfatase activity, incubation periods of between 2 and 20 h have been utilised (Table 2). As high throughput analysis is necessary for sports testing, especially during competitions, the long incubation times for hydrolysis can make their ability to contribute to a screening method unfeasible.

Hydrolysis is also pH-dependent and varies depending on the enzyme preparation utilised. The concentration of the conjugates present in the sample can affect the pH and thus can vary from urine to urine [18]. The pH of common hydrolysis conditions is generally slightly acidic to neutral as androgen glucuronides are more resistant to hydrolysis under alkaline conditions [27]. The conjugate moiety also plays a role with *H. pomatia* requiring an optimum pH 4.5–5.0 for β-glucuronidase and pH > 6.2 for sulfatase activity. The activity of *H. pomatia* is completely destroyed at pH 4.5 in acetate buffer [28].

The literature reveals that optimisation of the conditions are carried out one parameter at a time. However, this can lead to unreliable results as interactions between the conditions can occur. Using *H. pomatia*, *P. vulgata*, *Haliotis* entrails and Ketodase, a comparative study investigating the optimum hydrolysis conditions for DHEA, etiocholanolone and epitestosterone were performed using a response surface methodology [10]. The mathematical approach allowed for interactions between different conditions to be assessed and illustrated the complexity of determining suitable hydrolysis conditions. Using *Haliotis* entrails, hydrolysis was influenced by temperature and the amount of enzyme used. Interactions between temperature and time also affected hydrolysis. In contrast, a time–pH interaction existed for *P. vulgata* and was steroid and enzyme amount dependent. Effects could also be quadratic as with the case of pH for etiocholanolone or linear as observed for the pH effect on DHEA when using *H. pomatia*. The study concluded that *Haliotis* entrails were the best enzymatic preparation for the steroids investigated. Despite the influence that the conditions for hydrolysis can have on efficient cleavage, there have been publications which do not state the supplier nor conditions for enzyme hydrolysis [29].

In addition to the ambiguity over the enzyme choice and the conditions for enzymatic hydrolysis, there is also disparity over whether to hydrolyse pre- or post-extraction from urine. Originally, extraction followed by hydrolysis was the preferred approach but the majority of more recent studies favour direct hydrolysis (pre-extraction) on the urine (Table 2). However, direct hydrolysis has been shown to lead to incomplete hydrolysis or decreased recovery of the steroids of interest. Incomplete hydrolysis has been observed for androsterone and etiocholanolone glucuronides and this inefficiency, which was irrespective of the enzyme preparation used (Ketodase, *E. coli* or *H. pomatia*), and appeared to be related to undefined inhibitors present in the urine [30]. Cleavage of the sulfate moiety can be adversely affected owing to phosphate and sulfate ions inhibiting steroid sulfatase [19]. Bacteria may also metabolise some of the analytes during the enzyme incubation step [31]. Removal of interfering compounds by extraction followed by hydrolysis can lead to improved hydrolysis even when using less enzyme [25]. This is particularly pertinent with DHEA, with a balance between sufficient enzyme preparation for complete cleavage of the sulfate moiety but not to such an excess as to give insufficient recovery of DHEA.

As many sulfate conjugates are resistant to enzyme hydrolysis or generate unwanted by-products, the less specific chemical hydrolysis as an alternative to the sulfatase enzyme has been introduced. Chemical hydrolysis can be used either in combination with an enzyme preparation (commonly *E. coli*) or on an individual basis. Traditionally, chemical hydrolysis was achieved using hot acid with cleavage of the conjugate being strongly influenced by the choice of acid (hydrochloric or sulfuric), acid molarity, temperature and duration of the reaction [32]. More recently, chemical hydrolysis has been carried out by solvolysis and when compared to hot acid hydrolysis, leads to superior steroid recovery [33]. Solvolysis can utilise ethyl acetate in acidic conditions and heating for anything between 1 h at 55 °C [26], and 24–48 h at 37 °C [18,34]. Alternatively, methanolysis which is a variation of solvolysis using trimethylchlorosilane (TMCS) in methanol has been employed for a wide range of steroid androgens (Table 3). Cleavage of both the glucuronide and sulfated conjugates occurs due to the generation of hydrochloric acid from TMCS which then promotes hydrolysis [5].

Chemical hydrolysis is not without issues and may cause degradation of some analytes, increased levels of co-extractants, and increased matrix interference from degradation of macromolecules [35]. To remove matrix effects, chemical hydrolysis is performed following extraction of the steroid conjugates from the urine. Acid hydrolysis applied directly to urine is particularly undesirable leading to the formation of compounds from the action of the acid on the organic urine components [36]. Hot acid is generally now considered unreliable, in particular for DHEA sulfate due to low recoveries caused by steroid decomposition [37,38]. However, a recent study utilised hot acid in a one step hydrolysis-derivatisation procedure for DHEA sulfate (recovery not reported) [39].

To summarise, the issues with efficient sulfate hydrolysis has led to a focus on glucuronide-only analysis utilising *E. coli* which does not form unwanted by-products. For sulfate hydrolysis or samples containing both moieties, the choice of method is influenced by the steroids of interest. One of three approaches has been employed:

1. Enzyme hydrolysis using *H. pomatia*,
2. Chemical hydrolysis by solvolysis, or
3. Combination of *E. coli* followed by solvolysis.

However, there is no consensus over the conditions for either enzyme or chemical hydrolysis and to date; no universal method for steroid deconjugation is available.

## 3 Derivatisation

Following hydrolysis and extraction, urinary steroids require derivatisation prior to analysis by GC to improve their volatility, thermal stability and peak shape thus enhancing separation and detection. Most importantly, this approach means that it is not the conjugate or free steroid that is actually determined but rather a chemically modified form. Despite this disadvantage and the fact that derivatisation is considered to be a laborious task, GC/MS(/MS) is one of the most widely used methods for measuring steroids originally present in urine as conjugates [40]. Derivatisation of steroids can be carried out using silyation or acylation reactions, depending on the individual properties of the steroid and detection system. The choice of derivatisation mixture is dependent on the steroids of interest, as co-elution of the derivatised steroids may occur [41].

Silyation is the preferred steroid derivatisation approach for GC/MS with a variety of trimethylsilyating (TMS) reagents being available. The most popular are the *N*,*O*-bis(trimethylsilyl)-trifluoroacetamide (BSTFA) and the more volatile *N*-methyl-*N*-trifluoroacetamide (MSTFA). The use of catalysts is very common in the silylation process and necessary for tertiary hydroxyl groups and enolisation of the carbonyl function [42]. Enolisation of the keto group can be achieved by acylation with acid anhydride or silylation combining a silylating reagent and a catalyst such as potassium acetate or the more traditional trimethyliodosilane (TMIS) [43]. As TMIS is highly sensitive to hydrolysis and decomposition by oxygen and light, a reduction agent (ethanethiol, dithiothreitol or 2-mercaptoethanol) is often added to minimise iodine formation and postpone degradation of the derivatisation mixture.

A derivatisation procedure using MSTFA with ethanethiol and ammonium iodide (as an alternative to TMIS) suitable for GC/MS analysis has been routinely used by the WADA-accredited laboratories for athletic drug testing [44]. However, this derivatisation mixture may lead to the formation of ethyl thio adducts which can cause interpretation problems in sample analysis [41]. The failure of some steroids to provide a single reaction product, together with the chemical rearrangement of others, has been cited as a hindrance to official testing laboratories [45].

Whereas silyation has been the accepted precursor step for GC/MS(/MS), silyation reagents are considered incompatible with GC/C/IRMS due to deposition of silicon on the copper wire resulting in incomplete combustion. Acetylation is the standard derivatisation approach for GC/C/IRMS, with the derivatised products being very stable and exhibiting good chromatographic properties. The consequence of derivatisation for GC/C/IRMS determination is the addition of carbon atoms at each derivatised site. The fewer carbons added the less impact on the overall carbon isotope ratio. For acetylation, only two carbon atoms are added compared to three for silylation, due to derivatisation of just the hydroxyl groups and not the enols [46]. However one study identified incomplete derivatisation resulting in mixtures of non-, mono- and diacetylated steroids [47]. Consequently, the study evaluated the derivatisation mixture of MSTFA, dithiothreitol and TMIS on etiocholanolone, epiandrosterone, epitestosterone and DHEA. Poor reproducibility for DHEA using TMIS was observed with variation in the number of TMS groups but replacing TMIS with ammonium iodide gave well-resolved chromatographic signals, no peak tailing, reproducible measurements and a good signal-to-noise ratio [47].

## 4 LC/MS(/MS) and new techniques

**Fig. 3:**
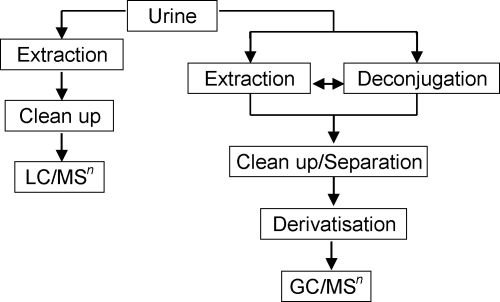
Overview of analytical methodologies for steroid determination.

In addition to the issues raised above, the hydrolysis and derivatisation steps also add time and expense to the overall methodology. To alleviate these problems and more importantly allow analysis of steroid sulfates without the problems associated with hydrolysis, an alternative analytical approach has gained popularity. LC/MS(/MS) allows for direct determination of the steroid conjugate, negating modification of the analyte structure to render it suitable for analysis (Fig. 3). Without the need for hydrolysis or derivatisation, the sample preparation time is greatly reduced leading to a reduction in analyte losses [48]. A review of the recent literature illustrates that steroid sulfate determination is important to the anti-doping field and the use of LC/MS(/MS) is a valuable tool to achieving this goal [49–52]. However, though LC/MS(/MS) may be utilised, hydrolysis of glucuronide and sulfated conjugates may still be employed [26].

LC/MS(/MS) is not, however, completely infallible and can suffer from ionisation and matrix effects leading to poor responses in conjugate quantification [53–56]. In addition and of particular relevance to the anti-doping field, whilst LC/MS can be used in place of GC/MS for determining the steroid profile, elucidating whether the steroid is exogenous or endogenous is still the premise of GC/C/IRMS.

Recently, a preparatory technique which renders steroid conjugates suitable for GC analysis without the need for hydrolysis or derivatisation has been developed. Originally utilised for liberating organic matter from petroleum source rocks [57], hydropyrolysis involves stripping the functional groups from steroids whilst faithfully retaining the carbon skeleton and its stereochemistry [58]. This approach has now been applied to fatty acids and free and conjugated steroids with determination by GC/C/IRMS [58–60].

## 5 Conclusions

The anti-doping field has many tools for analysing steroids relevant to substance abuse. The primary analytical tool is GC coupled to MS or IRMS as advocated by WADA. The preparative approaches of hydrolysis and derivatisation are a necessary prerequisite for GC determination. Unfortunately, the choice of hydrolysis is dependent on the steroid and conjugate moiety. Even then, the hydrolysis conditions are not defined and are often inefficient. Derivatisation also lacks a universal approach and can lead to chemical rearrangements and multiple reaction products. These issues have limited the focus to steroids conjugated with a glucuronide moiety. Though determining steroid sulfates continue to be of interest in the anti-doping field, the current direction for analysing the sulfated conjugates is by LC/MS(/MS) which alleviates the need for hydrolysis or derivatisation. Determining whether the steroidal origin is endogenous or exogenous is of vital importance and necessitates GC/C/IRMS. A new preparatory technique termed hydropyrolysis exhibits the potential to analyse both glucuronide and sulfated conjugates without the need for hydrolysis or derivatisation. By removing the functional groups to leave a carbon skeleton, its amenability to GC/C/IRMS analysis will allow conclusive determination of the origin of steroids.

## Figures and Tables

**Table 1:** Several issues arising from conjugate hydrolysis.

Issue	Example	References
Incomplete deconjugation	*H. pomatia* and *P. vulgata* unable to deconjugate androsterone-3-sulfate and testosterone-17-sulfate	[32]

Steroid conversion/decomposition	Increase in testosterone with *H. pomatia* from conversion of androst-5-ene-3β,17β-diol	[34]
	DHEA to epiandrosterone when using solvolysis	[61]
	Decrease in DHEA which is converted to androst-4-ene-3,17-dione with *H. pomatia*	[22]
	Hot acid hydrolysis causes steroid decomposition, resulting in low recoveries	[37]

Hydrolysis conditions	DHEA sulfate deconjugation favours higher temperatures	[19]
	Decrease in androsterone and etiocholanolone recoveries using higher incubation temperatures for *H. pomatia*	[18]
	The sulfatase activity of *H. pomatia* is completely destroyed at pH 4.5 in acetate buffer	[28]

Hydrolysis step in overall methodology	Inhibitors present in the urine led to incomplete hydrolysis of androsterone and etiocholanolone glucuronides	[30]
	Phosphate and sulfate ions inhibit steroid sulfatase	[19]
	Bacteria in the urine can metabolise steroids	[31]

**Table 2:** Recent biological (enzyme) deconjugation approaches as part of the analytical method.

Analyte	Enzymatic preparation	Conditions for deconjugation	Analytical procedure	References
19-Norandrosterone	*Escherichia coli* (K12 EC 3.2.1.31 Roche) G activity not stated	Adjusted to pH 6.0, 50 μl *E. coli* added then incubated at 40 °C for 1 h.	Urine → IS addition → hydrolysis → L–L extraction → LC separation + fractionation → derivatisation → GC/MS	[44]

Testosterone, epitestosterone, androstenedione, DHEA, androsterone, etiocholanlone	*E. coli* (K12 Boehringer Manneheim) G = 200 IU ml^−1^	125 ml of phosphate buffer (pH 7, 0.2 M) added to 2 ml urine. 50 μl *E. coli* added and incubated at 50 °C for 1 h.	Urine → IS addition → hydrolysis → L–L extraction → derivatisation → GC/MS	[62] following method in [63]

19-Norandrosterone, 19-noretiocholanolone, 19-norepiandrosterone	*E. coli* (Sigma), G activity not stated	1 ml phosphate buffer (0.1 M, pH 6.9) and 50 μl *E. coli* added and incubated at 50 °C for 1 h.	Urine → IS addition → SPE C18 → hydrolysis → chemical hydrolysis → L–L extraction → derivatisation → GC/MS	[14]

Testosterone, epitestosterone, androsterone, epiandrosterone, etiocholanolone, epietiocholanolone, dihydrotestosterone, DHEA, 5α-androstane-3α,17β-diol	*E. coli* (type VII-A Sigma), G = 5 × 10^3^ U ml^−1^	200 μl urine diluted with 800 μl potassium phosphate buffer (0.25 M, pH 6.9). 40 μl *E. coli* added and incubated at 37 °C for 22 h under gentle agitation.	Urine → IS addition → hydrolysis → L–L extraction →aqueous phase: SPE C18 → chemical hydrolysis → L–L extraction → LC/MS/MS, solvent phase: LC/MS/MS	[26]

Testosterone, DHEA, 5β-androstane-3α,17β-diol	*Helix pomatia* (G0876 Sigma), G and S activity not stated	10 ml 0.05 M acetate buffer (pH 5.5) added. 250 μl *H. pomatia*, incubated 55 °C for 3 h	Urine → L–L extraction → SPE C18 → hydrolysis → L–L extraction → SPE silica → LC separation + fractionation → derivatisation → GC/C/IRMS + GC/MS	[46]

Testosterone	*Helix pomatia* (Biosepra) G = 10^5^ FU ml^−1^, S = 10^6^ RU ml^−1^	2 ml acetate buffer (pH 5.2) to 5 ml urine. 25 μl *H. pomatia*, incubated at 55 °C for 2 h	Urine → IS addition → hydrolysis → SPE C18 → L–L extraction → SPE oasis → derivatisation → GC/MS	[64]

Epitestosterone, etiocholanolone, DHEA	Helix pomatia* (H-5 Sigma), G = 4–6 × 10^5^ U g^−1^, S = 15–40 × 10^3^ U g^−1^	Adjusted to pH 5.2, incubated at 50–52 °C for 20 h with 12,000 U of enzyme preparation.	Urine → IS addition → hydrolysis → SPE C18 → silica gel purification → ES addition → derivatisation → GC/MS. (* Also tested *H. pomatia* from Biosepra but enzyme activity not stated.)	[10]
	*Patella vulgata* (Sigma), G = 1–3 × 10^6^ U g^−1^, S = not determined	Adjusted to pH 6.8, incubated at 55 °C for 20 h with 12,000 U of enzyme preparation		
	*Haliotis* (abalone entrails) (Sigma) G = 4–8 × 10^5^ U g^−1^, S = 1–5 × 10^4^ U g^−1^	Adjusted to pH 5.2, incubated at 41 °C for 20 h with 12,000 U of enzyme preparation		
	Ketodase (Type B-1 Sigma), G = 5 × 10^5^ U g^−1^	Adjusted to pH 5.2, incubated at 50–52 °C for 1 h with 12,000 U of enzyme preparation		

Epitestosterone, etiocholanolone, DHEA	*Helix pomatia* (Biosepra) G = 10^5^ FU ml^−1^, S = 10^6^ RU ml^−1^	5 ml of 2 M acetate buffer (pH 5.2) added to 50 ml urine. 250 μl *H. pomatia*, incubated at 52 °C for 20 h.	Urine → hydrolysis → SPE C18 → L–L extraction → silica gel purification → LC separation + fractionation → ES addition → derivatisation → GC/C/IRMS and GC/MS	[65]
	*Haliotis* (abalone entrails) (Sigma), G = 4 × 10^5^ U g^−1^, S = 1–5 × 10^4^ U g^−1^	4.2 ml of 0.2 M acetate buffer (pH 5.2) added to 50 ml urine with 800 μl abalone entrails, incubated at 42 °C for 20 h.		[66]

DHEA, 5β-androstane-3α,17β-diol, androsterone, 5α-androstane-3α,17β-diol, etiocholanolone	*Escherichia coli* (Boehringer Manneheim) G = 80 U ml^−1^	Glucuronides–25 μl of *E. coli*, incubated at 50 °C for 1 h.	Urine → SPE C18 →anion exchange fractionation → sulfate hydrolysis → glucuronide hydrolysis → IS addition → L–L extraction → derivatisation → GC/C/IRMS	[12]
	*Ampullaria* (Nippon Bio-test), G = 4.2 × 10^4^ FU ml^−1^, S = 2.1 × 10^4^ RU ml^−1^	Sulfates–30 μl of *Ampullaria*, incubated at 60 °C for 1 h.		

DHEA: dehydroepiandrosterone; ES: external standard; FU: Fishman unit; G: glucuronide; GC/MS: gas chromatography/mass spectrometry; GC/C/IRMS: gas chromatography/combustion/isotope ratio mass spectrometry; IS: internal standard; IU: international unit; L–L: liquid–liquid; LC: liquid chromatography; S: sulfate; SPE: solid phase extraction; U: unit; RU: Roy unit.

**Table 3:** Recent chemical deconjugation approaches as part of the analytical method.

Analyte	Hydrolysis preparation	Conditions for deconjugation	Analytical procedure	References
19-Norandrosterone, 19-noretiocholanolone, 19-norepiandrosterone	Solvolysis (methanolysis)	1 ml TMCS solution (1 M TMCS in methanol) was added to dried residue and heated at 50 °C for 1 h.	Urine → IS addition → SPE C18 → enzyme hydrolysis → chemical hydrolysis → L–L extraction → derivatisation → GC/MS	[14]

DHEA, androsterone, etiocholanolone	Solvolysis (methanolysis)	10% TMCS added to dry steroid sulfate residue, heated at 50 °C for 30 min then cooled at room temperature.	Urine → SPE PADII resin → ion pairing extraction → SPE XAD-7 → chemical hydrolysis → hexane extraction → derivatisation → GC/MS	[7]

Testosterone, epitestosterone, epiandrosterone, androsterone, etiocholanolone, dihydrotestosterone, DHEA, 5α-androstane-3α,17β-diol, 5β-androstane-3α,17β-diol	Solvolysis (methanolysis)	1 ml TMCS solution (1 M TMCS in methanol) was added to dried residue and heated at 55 °C for 1 h.	Urine → IS addition → SPE C18* → hydrolysis → L–L extraction → derivatisation → GC/MS. (*SPE eluate divided into two parts, one for methanolysis and other for enzyme hydrolysis using *E. coli*.)	[5,67]

Testosterone, epitestosterone, androsterone, epiandrosterone, etiocholanolone, epietiocholanolone, dihydrotestosterone, DHEA, 5α-androstane-3α,17β-diol	Solvolysis	5 ml ethyl acetate/H_2_SO_4_ (250 ml/200 mg, 98%) added to 1 ml SPE eluate. Heated for 1 h at 55 °C under mild agitation.	Urine → IS addition → hydrolysis → L–L extraction →aqueous phase: SPE C18 → chemical hydrolysis → L–L extraction → LC/MS/MS, solvent phase: LC/MS/MS	[26]

DHEA	Hot acid hydrolysis (combined with derivatisation step)	50 μl each of acetone, acetic anhydride and acetic acid added to dried residue. Heated at 100 °C for 3 h.	Urine → SPE C18 → enzyme hydrolysis (*E. coli*) → SPE C18 → chemical hydrolysis + derivatisation → SPE C18 → GC/MS	[39]

Androsterone, etiocholanolone, DHEA	Hot acid hydrolysis (combined with solvent extraction)	10 ml benzene and 2 ml HCl added to dried residue. Refluxed at 80–83 °C for 20 min.	Urine → IS addition → L–L extraction → hydrolysis + solvent extraction → derivatisation → GC–FID	[36]

DHEA: dehydroepiandrosterone; FID: flame ionisation detector; GC/MS: gas chromatography/mass spectrometry; GC/C/IRMS: gas chromatography/combustion/isotope ratio mass spectrometry; IS: internal standard; L–L: liquid–liquid; LC: liquid chromatography; SPE: solid phase extraction; TMCS: trimethylchlorosilane.
